# Validity of diagnoses, procedures, and laboratory data in Japanese administrative data

**DOI:** 10.1016/j.je.2016.09.009

**Published:** 2017-01-27

**Authors:** Hayato Yamana, Mutsuko Moriwaki, Hiromasa Horiguchi, Mariko Kodan, Kiyohide Fushimi, Hideo Yasunaga

**Affiliations:** a Department of Clinical Epidemiology and Health Economics, School of Public Health, The University of Tokyo, Tokyo, Japan; b Department of Clinical Data Management and Research, Clinical Research Center, National Hospital Organization Headquarters, Tokyo, Japan; c Quality Management Center, Medical Hospital, Tokyo Medical and Dental University, Tokyo, Japan; d Department of Health Policy and Informatics, Tokyo Medical and Dental University, Graduate School of Medicine, Tokyo, Japan

**Keywords:** Administrative data, Validation, Diagnosis, Procedure, Laboratory data

## Abstract

**Background:**

Validation of recorded data is a prerequisite for studies that utilize administrative databases. The present study evaluated the validity of diagnoses and procedure records in the Japanese Diagnosis Procedure Combination (DPC) data, along with laboratory test results in the newly-introduced Standardized Structured Medical Record Information Exchange (SS-MIX) data.

**Methods:**

Between November 2015 and February 2016, we conducted chart reviews of 315 patients hospitalized between April 2014 and March 2015 in four middle-sized acute-care hospitals in Shizuoka, Kochi, Fukuoka, and Saga Prefectures and used them as reference standards. The sensitivity and specificity of DPC data in identifying 16 diseases and 10 common procedures were identified. The accuracy of SS-MIX data for 13 laboratory test results was also examined.

**Results:**

The specificity of diagnoses in the DPC data exceeded 96%, while the sensitivity was below 50% for seven diseases and variable across diseases. When limited to primary diagnoses, the sensitivity and specificity were 78.9% and 93.2%, respectively. The sensitivity of procedure records exceeded 90% for six procedures, and the specificity exceeded 90% for nine procedures. Agreement between the SS-MIX data and the chart reviews was above 95% for all 13 items.

**Conclusion:**

The validity of diagnoses and procedure records in the DPC data and laboratory results in the SS-MIX data was high in general, supporting their use in future studies.

## Highlights

• Validity of DPC and SS-MIX data was tested using chart review results as references.
• Specificity of DPC diagnoses was high; sensitivity was low and varied by conditions.
• Procedure records in DPC data were highly sensitive and specific.
• Accuracy of SS-MIX laboratory data exceeded 95% in all 13 items examined.

## Introduction

Administrative databases are widely used in medical research studies.^1^, ^2^, ^3^, ^4^ Their large sample size, population representativeness, and clinical information enables large-scale studies to be conducted inexpensively.^5^, ^6^ However, the use of administrative databases for research is based on an assumption that databases convey reasonably accurate data for health status and service utilization information. Because the use of inaccurate data could produce biased results,^6^, ^7^ validation of the information recorded in administrative databases is essential.

In previous validation studies, comorbidities were recorded with high specificity, but their sensitivities were low and variable across different diseases.^8^, ^9^, ^10^, ^11^, ^12^, ^13^ Studies have also shown that, despite accurate recording of major procedures, such as surgeries and invasive examinations, minor procedures not related to reimbursements were often under-reported.^14^, ^15^, ^16^ However, literature on validation studies is sparse compared with the widespread application of databases, and administrative database research has often used non-validated diagnostic or procedural codes.^17^

The Japanese Diagnosis Procedure Combination (DPC) database has been widely used in clinical epidemiology studies.^18^, ^19^, ^20^, ^21^ The DPC data are unique in that distinctions are made between main diagnosis, comorbidities, and complications, and unlimited numbers of procedures and medications can be recorded.^22^ In addition, the National Hospital Organization (NHO) introduced the Standardized Structured Medical Record Information Exchange (SS-MIX) standardized storage^23^ to its hospitals, enabling daily laboratory data to be recorded. However, there have been no validation studies for either the DPC or SS-MIX data.

The aim of the present study was to evaluate the validity of diagnoses, procedures, and laboratory results recorded as DPC and SS-MIX data. We conducted a multicenter validation study in NHO hospitals using chart review results as reference standards.

## Methods

### Data source

In Japan, the DPC-based lump-sum payment system was introduced in acute-care hospitals nationwide from 2003.^24^ The DPC data used for payments include patient demographics and selected clinical information, admission and discharge statuses, diagnoses, surgeries and procedures performed, medications, and special reimbursements for specific conditions. Diagnoses are recorded using International Classification of Diseases, Tenth Revision (ICD-10) codes by the attending physicians. Suspected diagnoses are allowed to be recorded, but are designated as such. There are six categories of diagnoses, each with a limited number of recordable diseases. One diagnosis each is coded for “main diagnosis”, “admission-precipitating diagnosis”, “most resource-consuming diagnosis”, and “second most resource-consuming diagnosis”. A maximum of four diagnoses each can be coded for “comorbidities present at time of admission” and “conditions arising after admission”. All procedures performed during hospitalization are recorded according to the Japanese fee schedule for reimbursement.

**Fig. 1:**
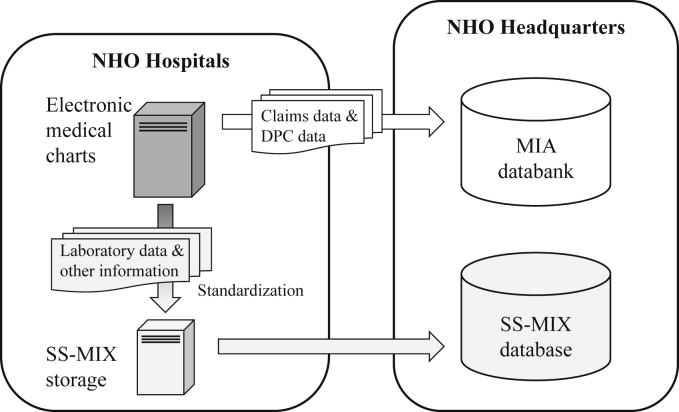
Flow of data in the National Hospital Organization. DPC, Diagnosis Procedure Combination; MIA, Medical Information Analysis; NHO, National Hospital Organization; SS-MIX, Standardized Structured Medical Record Information Exchange.

The NHO was established in 2004 to take over the management of the national hospitals. As of October 2014, there were 143 hospitals nationwide run by the NHO, including both general acute-care hospitals and specialized long-term-care hospitals. Fifty-four hospitals from 35 prefectures had implemented the DPC-based payment system, and the mean number of acute-care beds in these 54 hospitals was 410 (range, 135–730). All NHO hospitals provide administrative claims data to the Medical Information Analysis (MIA) databank, which is managed by the Clinical Research Center at NHO Headquarters. In NHO hospitals with implementation of the DPC-based payment system, the DPC data are also stored in the MIA databank. In addition, the NHO preliminarily introduced the SS-MIX standardized storage^23^ to its hospitals in 2013. The SS-MIX storage enables medical chart information from different vendors, including daily laboratory data, to be recorded in a standardized manner. In the SS-MIX storage, laboratory data are specified using JLAC-10 codes. The flow of data is shown in Fig. 1.

We conducted the present study on patients admitted to four acute-care NHO hospitals with implementation of both the DPC-based payment system and SS-MIX storage. The four hospitals were a 380-bed hospital in Shizuoka Prefecture, a 280-bed hospital in Kochi Prefecture, a 380-bed hospital in Fukuoka Prefecture, and a 420-bed hospital in Saga Prefecture. Laboratory data collected from the SS-MIX storage at each hospital and DPC data extracted from the MIA databank were compared with chart review results.

### Study population and variables

Among the patients aged ≥18 years on admission who were eligible for the DPC-based payment system and admitted and discharged between April 1, 2014 and March 31, 2015, we randomly selected 100 patients from each hospital, aiming to conduct 400 chart reviews in total.

The items examined in the study are presented in Table 1. The 17 diagnoses were diseases used for calculating the Charlson comorbidity index (CCI),^25^, ^26^, ^27^ which is widely used for risk adjustment in administrative database research studies. We also evaluated whether 10 specific procedures were performed on the day of admission. These procedures were selected from those used to calculate another index of severity, as identified by a previous study.^28^ We excluded blood examinations and drug use, as well as procedures that were rarely conducted (<5% among the 400 patients by searching the MIA databank before the chart reviews). The authors did not see the frequencies of the remaining 10 procedures until the chart reviews were complete. In addition, we examined the data of 13 laboratory tests performed on the day of admission. When multiple tests of the same item were conducted on the admission day, we selected the earliest one.

**Table 1:** Items examined in the study.

**Diagnoses**
Myocardial infarction
Congestive heart failure
Peripheral vascular disease
Cerebrovascular disease
Dementia
Chronic pulmonary disease
Rheumatic disease
Peptic ulcer disease
Mild liver disease
Diabetes without chronic complications
Diabetes with chronic complications
Hemiplegia or paraplegia
Renal disease
Any malignancy, including leukemia and lymphoma, except malignant neoplasm of skin
Moderate or severe liver disease
Metastatic solid tumor
AIDS/HIV
**Procedures [codes]**
Urine tests (general) [D000]
Urine microscopy [D002, D002-2]
Bacterial microscopy [D017]
Bacterial culture [D018]
Heart rate/respiration monitoring [D220]
Pulse oximetry [D223]
Radiography [E002]
Computed tomography scan [E200]
Peripheral intravenous infusion [G001]
Urinary catheter insertion [J063]
**Laboratory data**
White blood cell count
Platelets
Hemoglobin
Prothrombin time international normalized ratio
Na
Aspartate transaminase
Total bilirubin
Creatinine
Total cholesterol
C-reactive protein
Glucose
Hemoglobin A1c
Brain natriuretic peptide

AIDS, acquired immunodeficiency syndrome; HIV, human immunodeficiency virus.

### Chart reviews

We conducted chart reviews in the four hospitals from November 2015 through February 2016. Two authors (HY1 and MM) independently conducted chart reviews of the cases and identified whether patients had each of the 17 Charlson diseases, either as a primary diagnosis or a comorbidity present on admission. When chronic complications of diabetes were not documented, diabetes was classified as “diabetes without chronic complications”. The reviewers also identified whether each of the 10 procedures were performed on patients on the day of admission. When discrepancies arose, the two reviewers re-reviewed the charts and resolved the discrepancies through discussion. The inter-reviewer agreements for the 17 diseases before discussion in the chart reviews were evaluated using kappa coefficients and categorized as near-perfect (0.81–1.00), substantial (0.61–0.80), moderate (0.41–0.60), fair (0.21–0.40), and poor (0.00–0.20). Laboratory data were obtained by one author (MK) in one hospital and an assistant in three hospitals. The chief investigator (HY1) was consulted when questions arose about which laboratory data to use. The review process took 10–15 min per patient on average.

### Data extraction from databases

Patient demographics, diagnoses, and outcomes were extracted from the MIA databank. The 17 Charlson diseases were identified using the coding algorithms of Quan et al.^27^ A diagnosis was considered primary when it appeared as “main diagnosis” or “admission-precipitating diagnosis” and as a comorbidity when it appeared in “comorbidities present on admission”. Suspected diagnoses were excluded. In addition to the diagnoses, we searched for metastatic and recurrent malignancies using the TNM classification and primary/recurrent malignancy information recorded as clinical information. Records of procedures performed on the day of admission were extracted from the MIA databank using the codes presented in Table 1.

### Statistical analysis

The frequencies of the 17 Charlson diseases (as either a primary disease or a comorbidity) were identified by the chart reviews and DPC data. The sensitivity, specificity, positive predictive value (PPV), and negative predictive value (NPV) of the DPC data were calculated, accepting the chart review results as the reference standard. In addition to the original diagnoses, we combined the following diagnoses and assessed the resulting validity: diabetes (with or without chronic complications) and liver disease (mild, moderate, or severe). We also tested the validity of identifying metastatic tumors using clinical information in the DPC data (M1 in the TNM classification or recurrent cancer). The CCI values were calculated from the 17 diagnoses using the newly validated weight assignment by Quan et al,^29^ and the changes in the CCIs obtained using the different data sources were assessed.

The overall false-positive and false-negative cases for the 17 Charlson diseases in this study were defined as those with one or more false-positive diagnoses and one or more false-negative diagnoses, respectively. To evaluate the effect of recordable number of comorbidities in the DPC data, we categorized patients by the number of comorbidities recorded in the DPC data (0–3 vs. 4) and compared the overall false-positive and false-negative rates between the two groups.

Furthermore, we evaluated the validity of the primary diagnosis in the DPC data for identifying the Charlson diseases. Here, sensitivity was defined as the proportion of patients with one of the 17 Charlson diseases as the primary diagnosis by chart review who had the same diagnosis recorded in the DPC data. Specificity was defined as the proportion of patients without any of the 17 diseases as the primary diagnosis who also had none of these diseases recorded as the primary diagnosis in the DPC data.

The sensitivity, specificity, PPV, and NPV of the procedure records in the DPC data were calculated in a similar manner to the diagnoses, placing the chart review results as the reference standard. Laboratory data in the SS-MIX data were considered accurate if the value recorded in the SS-MIX data equaled that in the chart review, or if the SS-MIX data correctly identified that no testing of the item was conducted on the admission day.

Comparisons of categorical variables were conducted using the chi-square test, and a two-sided *P* value of <0.05 was considered significant. Statistical analyses were performed using IBM SPSS for Windows, version 21.0 (IBM Corp., Armonk, NY, USA).

### Standard protocol approvals, registrations, and patient consent

The study protocol was approved by the Central Ethics Review Board of the NHO, which deemed that written informed consent from participants was unnecessary. An announcement about the study and the possibility for participants to opt out of the study was made through the Internet.

## Results

### Overview and patient characteristics

We were able to conduct 80 chart reviews in three hospitals and 75 chart reviews in a fourth hospital (315 patients analyzed in total) due to time constraints. Cultures for methicillin-resistant *Staphylococcus aureus* were performed in most patients in one hospital, and vital signs could not be obtained from the charts of another hospital. Therefore, we excluded the former hospital from analyses of bacterial culture, and the latter hospital from analyses of heart rate/respiration monitoring and pulse oximetry. No patients had human immunodeficiency virus infection.

The mean age of the 315 patients was 66.8 (standard deviation [SD], 16.7) years, 183 (58.1%) were male, and 15 patients (4.8%) died during hospitalization. The inter-reviewer agreements for diagnoses before discussion by the reviewers are presented in Table 2. Agreement was near-perfect for six diagnoses, substantial for seven, moderate for two, and fair for one. Table 2 also presents the frequencies of 16 diseases in each hospital. Malignancy was the most commonly identified disease in all hospitals, and there was variation in disease frequencies across the four hospitals.

**Table 2:** Frequency of Charlson diseases and inter-reviewer agreements of identification in chart reviews.

Diagnoses	Hospital A (n = 80)		Hospital B (n = 80)		Hospital C (n = 75)		Hospital D (n = 80)		All hospitals (n = 315)		Inter-reviewer agreement (kappa)
	n	%	n	%	n	%	n	%	n	%	
Myocardial infarction	5	6.3	1	1.3	11	14.7	6	7.5	23	7.3	0.86
Congestive heart failure	12	15.0	2	2.5	7	9.3	11	13.8	32	10.2	0.82
Peripheral vascular disease	9	11.3	1	1.3	10	13.3	9	11.3	29	9.2	0.71
Cerebrovascular disease	11	13.8	7	8.8	5	6.7	15	18.8	38	12.1	0.61
Dementia	7	8.8	2	2.5	3	4.0	4	5.0	16	5.1	0.83
Chronic pulmonary disease	5	6.3	5	6.3	12	16.0	5	6.3	27	8.6	0.82
Rheumatic disease	1	1.3	2	2.5	1	1.3	3	3.8	7	2.2	0.93
Peptic ulcer disease	2	2.5	1	1.3	5	6.7	1	1.3	9	2.9	0.62
Mild liver disease	4	5.0	7	8.8	3	4.0	8	10.0	22	7.0	0.56
Diabetes without chronic complications	10	12.5	10	12.5	9	12.0	17	21.3	46	14.6	0.76
Diabetes with chronic complications	6	7.5	0	0.0	6	8.0	5	6.3	17	5.4	0.63
Hemiplegia or paraplegia	6	7.5	2	2.5	3	4.0	3	3.8	14	4.4	0.25
Renal disease	4	5.0	3	3.8	5	6.7	3	3.8	15	4.8	0.46
Malignancy^a^	16	20.0	25	31.3	28	37.3	28	35.0	97	30.8	0.83
Moderate or severe liver disease	0	0.0	4	5.0	1	1.3	1	1.3	6	1.9	0.66
Metastatic solid tumor	7	8.8	9	11.3	12	16.0	13	16.3	41	13.0	0.71

a Includes leukemia and lymphoma, excludes malignant neoplasm of skin.

### Validity of diagnosis

The frequencies of the Charlson diseases and validity indices for the DPC data are presented in Table 3. With the exception of peptic ulcer disease, the frequencies were lower in the DPC data than in the chart reviews. Sensitivity ranged from 0% (hemiplegia or paraplegia) to 83.5% (malignancy) and was lower than 50% for seven of 17 diseases, while specificity was above 96% for all diseases. PPV ranged from 23.1% (peptic ulcer disease) to 100% (dementia and moderate or severe liver disease, both with specificity of 100%), and exceeded 80% for 9 diseases. NPV was above 90% for all diseases.

**Table 3:** Frequencies of diagnoses and validity indices for the DPC data-based diagnosis identification.

Diagnoses	Frequency (charts)		Frequency (DPC data)		Sensitivity (%)	Specificity (%)	PPV (%)	NPV (%)
	n	%	n	%				
**Original Charlson diseases identified by recorded diagnoses**								
Myocardial infarction	23	7.3	13	4.1	52.2	99.7	92.3	96.4
Congestive heart failure	32	10.2	29	9.2	68.8	97.5	75.9	96.5
Peripheral vascular disease	29	9.2	12	3.8	34.5	99.3	83.3	93.7
Cerebrovascular disease	38	12.1	22	7.0	50.0	98.9	86.4	93.5
Dementia	16	5.1	6	1.9	37.5	100.0	100.0	96.8
Chronic pulmonary disease	27	8.6	18	5.7	33.3	96.9	50.0	93.9
Rheumatic disease	7	2.2	5	1.6	57.1	99.7	80.0	99.0
Peptic ulcer disease	9	2.9	13	4.1	33.3	96.7	23.1	98.0
Mild liver disease	22	7.0	13	4.1	36.4	98.3	61.5	95.4
Diabetes without chronic complications	46	14.6	33	10.5	52.2	96.7	72.7	92.2
Diabetes with chronic complications	17	5.4	6	1.9	29.4	99.7	83.3	96.1
Hemiplegia or paraplegia	14	4.4	0	0.0	0.0	100.0		95.6
Renal disease	15	4.8	10	3.2	53.3	99.3	80.0	97.7
Malignancy^a^	97	30.8	86	27.3	83.5	97.7	94.2	93.0
Moderate or severe liver disease	6	1.9	3	1.0	50.0	100.0	100.0	99.0
Metastatic solid tumor	41	13.0	28	8.9	58.5	98.5	85.7	94.1
**Combined Charlson diseases identified by recorded diagnoses**								
Liver disease (any)	28	8.9	15	4.8	39.3	98.6	73.3	94.3
Diabetes (any)	63	20.0	39	12.4	55.6	98.4	89.7	89.9
**Disease identified by clinical information**								
Metastatic tumor	41	13.0	41	13.0	73.2	96.0	73.2	96.0

DPC, Diagnosis Procedure Combination; NPV, negative predictive value; PPV, positive predictive value.

a Includes leukemia and lymphoma, excludes malignant neoplasm of skin.

When combining two conditions in two diagnoses (liver disease and diabetes), we observed modest increases in sensitivity and specificity compared with the original diagnoses that only included less severe conditions. Identification of metastatic tumors using clinical information in the DPC data had higher sensitivity with slightly lower specificity compared with diagnosis-based identification. The mean CCIs calculated from the chart review results and DPC data were 2.2 (SD, 2.9) and 1.5 (SD, 2.4), respectively. Of the 315 patients, the CCIs calculated from the two data sources were equal to each other in 198 cases (62.9%), lower in the DPC data in 89 cases (28.3%), and higher in the DPC data in 28 cases (8.9%).

Of the 315 patients, the number of diagnoses recorded as comorbidities present on admission was zero in 50 cases (15.9%), one in 53 cases (16.8%), two in 63 cases (20.0%), three in 43 cases (13.7%), and four in 106 cases (33.7%). Overall, there were 53 patients (16.8%) with false-positive diagnoses for at least one of the 17 diagnoses, and 128 (40.6%) with one or more false-negative diagnoses. Compared with the patients with 0–3 diagnoses recorded as comorbidities, the patients with four diagnoses recorded as comorbidities had a higher overall false-positive rate (27.4% vs. 11.5%, p < 0.001) and a higher overall false-negative rate (51.9% vs. 34.9%, p = 0.004).

According to the chart reviews, there were 123 patients (39.0%) with one of the 17 Charlson diseases as the primary diagnosis, and 192 patients (61.0%) without such a diagnosis. The sensitivity, specificity, PPV, and NPV of the primary diagnosis in the DPC data were 78.9% (97/123), 93.2% (179/192), 88.2% (97/110), and 87.3% (179/205), respectively.

### Validity of procedures

The frequencies of conducted procedures and validity indices for the DPC data are presented in Table 4. Sensitivity was low for four procedures: heart rate/respiration monitoring (66.7%), pulse oximetry (21.1%), peripheral intravenous infusion (72.7%), and urinary catheter insertion (65.5%). For the other six procedures, sensitivity exceeded 90%. The DPC data were also highly specific, with the lowest of the 10 procedures having a specificity of 88.5% (pulse oximetry).

**Table 4:** Frequencies of procedures and validity indices for the DPC data-based diagnosis identification.

Procedures	Frequency (charts)		Frequency (DPC data)		Sensitivity (%)	Specificity (%)	PPV (%)	NPV (%)
	n	%	n	%				
Urine tests (general)	74	23.5	77	24.4	98.6	98.3	94.8	99.6
Urine microscopy	35	11.1	36	11.4	97.1	99.3	94.4	99.6
Bacterial microscopy	35	11.1	32	10.2	91.4	100.0	100.0	98.9
Bacterial culture	34	14.5	33	14.0	97.1	100.0	100.0	99.5
Heart rate/respiration monitoring	30	12.8	36	15.3	66.7	92.2	55.6	95.0
Pulse oximetry	209	88.9	47	20.0	21.1	88.5	93.6	12.2
Radiography	161	51.1	158	50.2	97.5	99.4	99.4	97.5
Computed tomography scan	93	29.5	94	29.8	100.0	99.5	98.9	100.0
Peripheral intravenous infusion	165	52.4	122	38.7	72.7	98.7	98.4	76.7
Urinary catheter insertion	29	9.2	27	8.6	65.5	97.2	70.4	96.5

DPC, Diagnosis Procedure Combination; NPV, negative predictive value; PPV, positive predictive value.

### Validity of laboratory data

The agreements for laboratory data between the chart review results and the SS-MIX data are presented in Table 5. Based on the chart review results, the frequency of the conducted tests ranged from 18% (brain natriuretic peptide) to 63% (white blood cell count, hemoglobin, and creatinine). The SS-MIX data were accurate in over 95% of cases for all 13 tests examined.

**Table 5:** Accuracy of laboratory data recorded in the SS-MIX storage.

Laboratory data	Same results recorded in charts and SS-MIX		No result recorded in charts or SS-MIX		Different results recorded in charts and SS-MIX		Result recorded in charts only		Result recorded in SS-MIX only		Agreement between charts and SS-MIX (%)
	n	%	n	%	n	%	n	%	n	%	
WBC	195	61.9	115	36.5	3	1.0	1	0.3	1	0.3	98.4
Platelets	194	61.6	115	36.5	3	1.0	1	0.3	2	0.6	98.1
Hemoglobin	192	61.0	115	36.5	6	1.9	1	0.3	1	0.3	97.5
PT-INR	116	36.8	192	61.0	1	0.3	1	0.3	5	1.6	97.8
Na	190	60.3	121	38.4	2	0.6	1	0.3	1	0.3	98.7
AST	194	61.6	116	36.8	2	0.6	1	0.3	2	0.6	98.4
Total bilirubin	186	59.0	125	39.7	3	1.0	1	0.3	3	1.0	97.8
Creatinine	197	62.5	115	36.5	1	0.3	1	0.3	1	0.3	99.0
Total cholesterol	89	28.3	222	70.5	0	0.0	2	0.6	2	0.6	98.7
CRP	175	55.6	131	41.6	4	1.3	1	0.3	4	1.3	97.1
Glucose	130	41.3	172	54.6	1	0.3	1	0.3	11	3.5	95.9
Hemoglobin A1c	58	18.4	252	80.0	0	0.0	2	0.6	3	1.0	98.4
BNP	57	18.1	253	80.3	1	0.3	0	0.0	4	1.3	98.4

AST, aspartate transaminase; BNP, brain natriuretic peptide; CRP, C-reactive protein; PT-INR, prothrombin time international normalized ratio; SS-MIX, Standardized Structured Medical Record Information Exchange; WBC, white blood cell count.

## Discussion

In the present study, we evaluated the validity of diagnoses, procedures, and laboratory data recorded as administrative data, using chart review results as the reference standard. As with any administrative data, there were some limitations to the recorded data. However, our results suggest that the DPC and SS-MIX data can serve as relatively accurate substitutes for clinical data in future studies.

The specificity of DPC diagnosis records in identifying the Charlson diseases was high, but the sensitivity was low and varied across conditions. Within the diseases examined, there seemed to be considerable under-reporting, while coding of conditions that did not exist (i.e., up-coding) appeared uncommon. Compared with previous validation studies, the sensitivity was similar or slightly lower in the DPC data.^8^, ^9^, ^10^, ^11^, ^12^, ^13^ Two reasons could account for these present findings. First, attending physicians, who are not professional coders, are obliged to record the diagnoses and may not be aware of the specific codes used to express conditions. For example, “hemiplegia or paraplegia” may not be recognized as a diagnosis, and codes within “diabetes” (with or without chronic complications) may be disregarded. Second, only two primary diagnoses and up to four comorbidities can be recorded in the DPC data, in contrast to up to 16 diagnoses in a database in Canada.^8^, ^9^, ^10^, ^11^, ^12^ In the present study, patients with four recorded comorbidities had a higher overall false-negative rate compared with patients with three or less recorded comorbidities. Even when comorbidities are recognized, some of them may not be recorded when all four “slots” for comorbidities are occupied. Meanwhile, sensitivity (78.9%) and specificity (93.2%) were both high when limited to primary diagnosis.

A strength of the present study is the random selection of participants and inclusion of those without specific diseases, which enabled calculations of sensitivity and specificity. From our results, PPV and NPV can be estimated by assigning disease prevalences. Under random selection, the PPV was acceptably high for most diseases, thus warranting diagnosis-based patient identification in future studies. By limiting the study population to patients with higher disease prevalences (e.g., elderly patients), the PPV would be higher. However, it should also be noted that patients identified using database diagnoses may be an underestimated or unrepresentative sample of the disease population.

When we calculated the CCIs, the value derived from the DPC data tended to be lower than that derived from the chart review results. There were differences in the CCIs for 37.1% of the patients, including 28.3% with lower values when using the DPC data, which is consistent with previous studies.^9^, ^10^ An international comparison of databases also showed variation in the ability of the CCI to predict mortality,^29^ which may partly arise through the characteristics of databases. Although the CCI is a commonly used index of comorbidity, its interpretation requires caution because the values may be biased compared with clinical studies or studies using different databases.

Cancer classifications were among the clinical information stored in the DPC data, and we tested their ability to correctly identify clinical conditions. A simple method of using M1 or recurrent tumor was accurate in identifying metastatic tumors (sensitivity: 73.2%; specificity: 96.0%). Such clinical information may serve as a useful substitute for diagnosis, especially when the number of recordable diagnoses is limited. Other clinical information in the DPC data included consciousness, New York Heart Association classification, and Hugh-Jones classification, which may also be used to identify clinical conditions.^30^

We also evaluated the validity of procedure records in the DPC data. Although the 10 procedures in our study were minor, the DPC data identified most of them with high accuracy. This is in contrast to a previous study, wherein the sensitivities for X-ray scans, computerized tomography scans, insertion of indwelling urinary catheter, and venous catheterization were 0%, 0.5%, 0%, and 39.6%, respectively.^14^ Only 10 procedures can be coded in the database used in the previous study, whereas there is no such limit in the DPC data. This difference could explain the different rates of under-reporting. Heart rate/respiration monitoring and pulse oximetry can only be reimbursed when patients have severe conditions under continuous monitoring, but such criteria were difficult to define in the chart reviews and we included all patients in whom these two procedures were performed. This could have resulted in the low sensitivities.

The laboratory data recorded in the SS-MIX storage had excellent agreement with the chart review results. Instead of summarizing patient information for use in payment or database construction, the SS-MIX storage is designed to standardize and store electronic medical records themselves.^23^ Although standardization of laboratory results using JLAC-10 codes in the newly-introduced SS-MIX system was considered a possible challenge, the data were collected accurately. Our previous study utilized the SS-MIX storage in its preliminarily introduced hospitals,^31^ but a large-scale study remains to be conducted. SS-MIX data would be a useful source for future clinical epidemiology studies.

Several limitations of the present study should be noted. First, we used chart review results as the reference standard to assess the validity of the DPC and SS-MIX data. These reviews are dependent on the quality of the charts, and unrecorded information could not be captured. An ideal reference standard should identify a condition that is truly present in a patient. However, such information is difficult to obtain, and previous studies have also regarded chart reviews as the best available references. Also, the kappa coefficients for inter-reviewer agreement were low for some diagnoses, which poses a challenge when considering the chart review results as a reference. Second, we conducted the study in four hospitals within the NHO. Although we confirmed variation in the disease prevalences across the hospitals, it remains unclear whether the results can be extrapolated to other institutions. Importantly, these hospitals were early adopters of the SS-MIX storage, and it is possible that their data collection and recording are more accurate compared with other hospitals. Furthermore, there could be some features of the NHO hospitals that make their recorded data more reliable compared with other hospitals in Japan. One such example is lectures to health information administrators that aim to improve disease coding. However, other hospitals could also be conducting similar initiatives. Third, we assessed limited numbers of diagnoses, procedures, and laboratory data, and the validity of items that were not examined cannot be determined. Last, the number of participants was small, and the sensitivity estimates may be statistically unstable for some diseases with low prevalence.

The present study adds to the sparse literature of studies validating administrative data and can serve as an important basis for future studies using DPC and SS-MIX data. The results support the usefulness of diagnoses and procedure records within the DPC data, provided that the investigators using these data acknowledge their limitations and make appropriate interpretations. We also confirmed the validity of laboratory data in the newly-introduced SS-MIX storage, and the SS-MIX storage would add a considerable amount of information to future database-based studies.

## Conflicts of interest

None declared.
